# Functional, Nutritional and Biological Properties of Ice Cream Produced by Yeast Inoculation

**DOI:** 10.1002/fsn3.71847

**Published:** 2026-05-14

**Authors:** Ayşe Janseli Denizkara, Mehmet Kilinç, Gökhan Akarca

**Affiliations:** ^1^ Faculty of Engineering, Food Engineering Department Afyon Kocatepe University Afyonkarahisar Turkey

**Keywords:** functional, ice cream, nutrition, probiotic, *S. boulardii*

## Abstract

The study evaluated the impact of three yeast strains ( *Saccharomyces cerevisiae* , probiotic *Saccharomyces cerevisiae* var. *boulardii*, and probiotic *Kluyveromyces marxianus* var. *lactis*) on the physicochemical, microbiological, textural, and nutritional properties of ice cream. Statistical analysis using ANOVA revealed that yeast inoculation significantly affected (*p* < 0.05) the product characteristics. *S. cerevisiae* led to the lowest first drip (20.78 s), complete melting time (86.78 s), overrun (44.80%), firmness (13.71 g), consistency (251.21 g s), and pH (6.30). Microbiological analysis immediately after production showed that *S. boulardii* exhibited the highest initial viability (8.05 log cfu/g), exceeding the probiotic threshold, while *S. cerevisiae* and *K. lactis* achieved 7.09 and 6.16 log cfu/g, respectively. Regarding nutritional profiles, *S. cerevisiae* significantly increased formic, malic, and lactic acids, and resulted in the highest saturated fatty acid content (78.48%). *S. boulardii* and *K. lactis* also modified the fatty acid and organic acid profiles, demonstrating strain‐dependent metabolic effects, although they generally produced milder physical alterations than *S. cerevisiae* . In conclusion, inoculating ice cream with these yeasts, particularly *S. boulardii*, successfully enhances its functional and nutritional value, offering a highly practical and promising technological approach for the dairy industry to develop novel, health‐promoting functional desserts.

## Graphical abstract

This study investigates the inoculation of ice cream mix with three different species of yeast. The results demonstrate that yeast inoculation significantly enhances the functional value, provides acquired probiotic properties, and improves the overall nutritional profile of the final ice cream product.

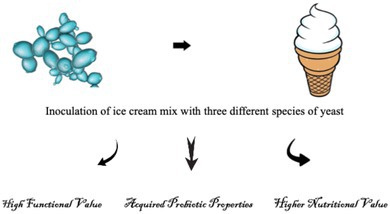

## 1 Introduction

Ice cream is a globally recognized food that can be consumed during meals as well as between meals (Genovese et al. 2022). Ice cream is a physicochemical food. It consists of fat globules, ice crystals, proteins, sugars, polysaccharides, salts, and air bubbles in a frozen concentrated solution (Akbari et al. 2019; Arslaner and Salik 2020). Excessive intake of ice cream, which is rich in nutrients like fat and sugar, might elevate the risk of health problems such as obesity and elevated cholesterol levels (Krystyjan et al. 2015). The adoption of a healthy lifestyle by consumers and their interest in new foods produced in this area have led to some concerns regarding ice cream. As a result, demands for products with lower calories and highlighted functional properties have emerged. Consequently ice cream manufacturers have also started to develop products in line with these demands (Díaz et al. 2020; Durmaz et al. 2020).

Probiotics are microorganisms (bacteria and yeast) that confer health advantages to the human host when ingested in sufficient quantities. Probiotic bacteria are defined as live microorganisms that, when administered in sufficient quantities, provide health benefits to the host. This fundamental concept has recently been expanded to include yeasts in addition to bacteria (Alkalbani et al. 2022). To be considered probiotics, they need to show certain traits, such as being able to survive the tough conditions in the gut, clumping together, acting like helpful bacteria, and not causing disease.

Studies have revealed the probiotic properties of the yeast species *S. boulardii* and *K. lactis* due to their survival throughout the gastrointestinal system and presence in functional matrices, adhesion to the intestinal mucosa and interaction with epithelial cells, antagonism against enteropathogens and inhibition of pathogen adhesion, immunomodulatory effects and modulation of host defense, and clinical and in vivo evidence of efficacy (Ryan et al. 2011; Łukaszewicz 2012; Arévalo‐Villena et al. 2018). Research involving these yeast species has demonstrated their efficacy in treating many gastrointestinal disorders induced by bacteria and viruses, including foodborne and travel‐related diarrhea. Moreover, the diverse therapeutic qualities of these strains are driving an increase in demand for nutritional supplements containing them within the probiotic industry (Czerucka et al. 2007; Feizizadeh et al. 2014; Profir et al. 2015). Probiotic yeasts can be easily used not only in beverages but also in the production of many foods (Swieca et al. 2019).

There is still no single definition for a functional food. Functional foods are those that contain components capable of positively influencing customers' health, and recently, the demand for such meals has surged significantly. Ice cream possesses significant potential for development as a functional food, driven by high consumer demand, and can be enhanced with fruits abundant in phenolics, probiotics, and prebiotics (Hernández‐Riveros et al. 2024). The protective functions of milk proteins, lipids, lactose, and other constituents enhance ice cream's efficacy as a probiotic carrier meal, as previously mentioned. The aim of developing functional ice cream is to have a product that is rich in nutritional value compared to traditional ice cream, helps protect human health, and can benefit people of all ages and social classes (Soukoulis and Tzia 2018; Munk et al. 2018; Hossain et al. 2020).

Ice cream is not conventionally classified as a fermented dairy product. It may be converted into a fermented functional food by inoculating the mixture with suitable microorganisms and permitting active microbial metabolism (fermentation) before freezing. The active fermentation method improves the product's functional, textural, sensory, and nutritional attributes (Akarca et al. 2024; Gao et al. 2021).

The majority of studies undertaken to date have concentrated on utilizing ice cream as a medium for probiotic *Lactobacillus* species. Nevertheless, there is a scarcity of studies that elucidate the characteristics of ice creams generated by inoculating probiotic yeast species (Göktaş et al. 2022).

The primary aim of this research was to examine the alterations in the physicochemical, textural, and nutritional characteristics of ice cream induced by active fermentation from two probiotic yeast species (*S. boulardii* and *K. lactis*) and one industrial yeast ( *S. cerevisiae* ). The primary hypothesis posited that yeast‐mediated fermentation would not only provide probiotic benefits by sustaining viable cell counts above the therapeutic threshold but also markedly improve the nutritional profile (including organic and fatty acid content) without detracting from the preferred physical quality of the ice cream.

## 2 Materials and Method

### 2.1 Materials

The raw cow's milk (dry matter: 11.41%; titratable acidity [% lactic acid]: 0.167; pH: 6.52; protein: 3.04%; fat: 3.56%) used in the research was obtained from a local producer in the Afyonkarahisar region. The salep and granulated sugar (sucrose from beets) were purchased at a nearby market in the same vicinity. The yeast strains used in this study, *Saccharomyces cerevisiae* (ATCC 9763), *Saccharomyces boulardii* (ATCC 74012), and *Kluyveromyces lactis* (ATCC 48793), were acquired commercially from the American Type Culture Collection (ATCC) in freeze‐dried form. Upon receipt, the strains were activated per the manufacturer's instructions and kept at 4°C in a refrigerator as stock cultures until used in ice cream manufacturing. To mitigate any variations in raw materials, milk, and kaymak from identical manufacturing batches were used in all experimental and control groups.

### 2.2 Yeasts Used in the Study

In this study, strains of *Saccharomyces cerevisiae* (ATCC 9763), *Saccharomyces boulardii* (ATCC 74012), and *Kluyveromyces lactis* (ATCC 48793) were used. The strains were stored in the refrigerator (4°C) until they were used in ice cream production.

### 2.3 Preparation of Yeast Strains

The yeasts utilized in production (*S. cerevisiae, S. boulardii*, and *K. lactis*) were generated by altering the methodology of Göktaş et al. (2022). Yeast strains were cultivated in Yeast Extract Rose Bengal Broth (Himedia, M955, USA) and incubated for 5 days at 25°C. Strains cultivated in a 2 L volume (Göktaş et al. (2022) cultivated the strains in a 5 L volume) to get the requisite concentrations before inoculating the ice cream mixture. Following the incubation periods, cultures were centrifuged at 7000 rpm for 10 min at 22°C, and the resulting pellets were washed twice with PBS (Phosphate Buffered Saline, Merck, 524650, Germany). Yeast strains were introduced into the pasteurized ice cream mixture at 37°C prior to the aging process. It has been confirmed thru microbiological analyzes that the yeast counts inoculated into the ice cream mix are in the range of 10^7^10^8^ log cfu/mL.

### 2.4 Ice Cream Production

**FIGURE 1:**
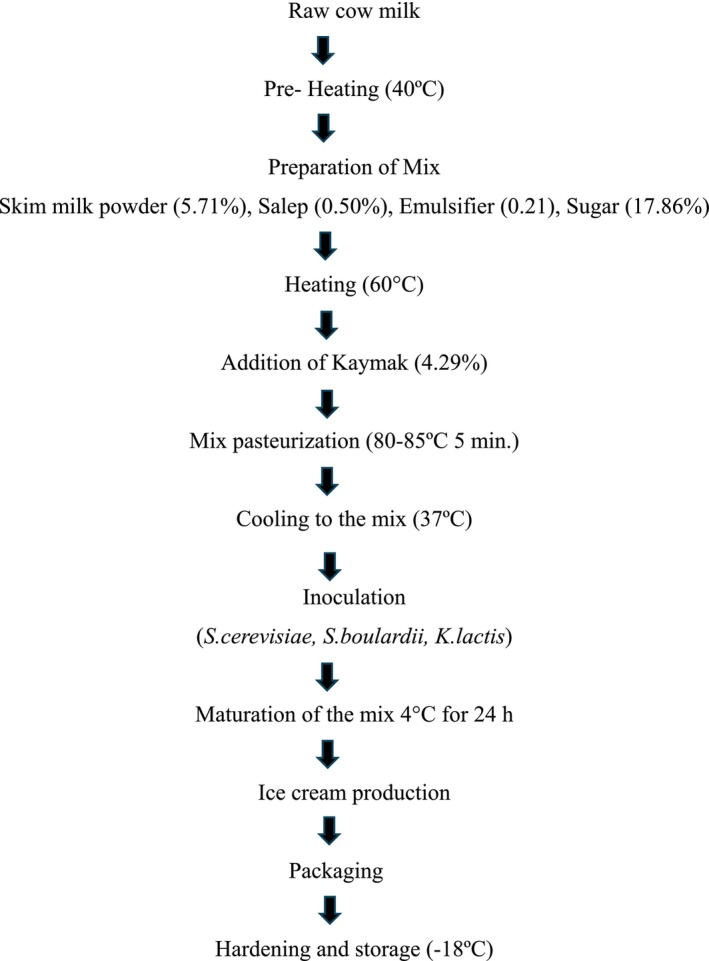
Ice cream production (per 2 kg ice cream sample).

Raw cow's milk was pre‐heated to 40°C, then an ice cream mix was prepared according to the formulation shown in Figure 1, followed by pasteurization (80°C–85°C for 5 min). After pasteurization, the mixture was cooled to 37°C (Kılınç et al. 2022; Akarca et al. 2024). The mixture was thereafter partitioned into equal 3 kg parts. To guarantee an adequate probiotic concentration, the activated pure cultures of the three distinct yeast strains (*S. cerevisiae, S. boulardii*, and *K. lactis*) were individually injected into each aliquot to attain a final goal concentration of 10^7^ to 10^8^ cfu/mL. Following the inoculation process, the ice cream mixes were matured at 4°C for 24 h (Göktaş et al. 2022). The resultant mixes were then transformed into ice cream at −5°C using an ice cream machine (CRM‐GEL 25C, Italy) (rotation speed: 75 rpm, the cooling temperature: −18°C, and the time: 4 min). Two hundred fifty grams of each mixture were put in sterilized glass containers and solidified at −24°C. The gathered materials were stored at −18°C until the analyses were completed (Figure 1). Each sample batch was produced in a quantity of 2 kg.

### 2.5 First Dripping Time

From the samples stored at −18°C before the test, 10‐g were placed on stainless steel wire sieves set on weighed glass containers and allowed to melt at 20°C. Then, the stopwatch was started, and the moment the ice cream began to melt and the first drops fell was recorded. The ambient temperature where the analyses were conducted was monitored with a digital thermometer. The measurements were taken in pairs in parallel (Akarca et al. 2024).

### 2.6 Meltdown Time

In a deep freezer (Uğur, UDD560 BK, Turkey) at −18°C, samples of hardened ice cream were taken and placed on a stainless‐steel wire grid with a wire width of 0.2 cm on a 500 mL glass beaker, where they were allowed to melt at 20°C, and a stopwatch was started. Then, the ice cream samples were left until they completely melted. The time taken for complete melting (in minutes) was recorded, and the total melting time was determined (Güven and Karaca 2002). The temperature of the environment where the analyses were conducted was monitored with a digital thermometer, and the analyses were performed in parallel pairs.

### 2.7 Overrun

Ice cream samples stored in a deep freezer at −18°C were first weighed and placed in a 500 mL glass measuring cylinder. Then, the same ice cream samples were placed in a 500 mL beaker and melted using a water bath. The liquid amalgam was transferred to a sterilized measuring cylinder of the same capacity and weighed again (Jimenez Florez et al. 1993). The temperature of the environment where the analyses were conducted was monitored with a digital thermometer, and the analyses were performed in parallel pairs.

### 2.8 Texture Analyses of Ice Cream Mix Samples

The texture values of the ice cream mixes (firmness, consistency, cohesiveness, and index of viscosity) were determined using a TA.XT Plus Texture Analyzer (Stable Micro Systems, Godalming, Surrey, UK) with back extrusion rig hardware (TA‐30A, 7.62 cm in diameter, cylindrical acrylic, and 10 mm in height).

According to the back‐extrusion technique, power‐time graphs were generated based on the positive areas when the probe was immersed in each sample and the negative areas when it was removed. Texture Exponent 32 (2007) software (Stable Micro Systems, Godalming, UK) was used to determine the textural properties of the samples. The evaluated parameters were defined as hardness (g) as the maximum positive force, consistency (gs) as the positive region area, cohesion as the maximum negative force, and viscosity index (gs) as the negative region area (Sert et al. 2017). The analyses were performed in pairs of parallel processes.

### 2.9 pH Value

Ice cream samples stored at −18°C were completely melted at 20°C in a temperature‐controlled environment. Next, samples were first mixed with sterilized pure water in a 1/10 ratio and then homogenized in a homogenizer (Daihan Wisestir, HS‐30 T, South Korea). Before the measurements, the pH meter was calibrated with three different buffer solutions. Then, the pH values were measured using a pH meter (Hanna, HI 2215 pH/ORP) (Akarca et al. 2024). pH analyses were performed in two parallel processes.

### 2.10 Water Activity (a_w_)

The water activity values of the ice cream samples were determined using a water activity analysis device (Novasina LabTouch‐aw, Lachen, Switzerland) (AOAC 2016). The temperature of the environment where the analyses were conducted was monitored with a digital thermometer, and the analyses were performed in parallel pairs.

### 2.11 Color Values (L*, a*, b*)

The color values of the samples were determined using a colorimeter (Minolta Co., Osaka, Japan) according to the Hunter color measurement system (Ruiz‐Gutiérrez et al. 2014). Color measurements were taken from five different points of the samples in a double parallel manner. The ambient temperature during the analyses was recorded using a digital thermometer.

### 2.12 Total Yeast Count

The determination of the count of yeasts added to the ice cream mixes was carried out using potato dextrose agar (Merck, 110130, Germany). Before the microbiological analyses, serial dilutions were prepared from the samples using 0.1% buffered peptone water (Merck, 107228, Germany), and the analyses were conducted according to the spread plate method. The Petri dishes that were inoculated were incubated under aerobic conditions at 25°C for 5–7 days in an incubator (Campos and Cristianini 2007). Analyses were performed in a sterile culture chamber (Crystal nova pro, Korea) under controlled atmosphere conditions, using a double parallel sampling method.

### 2.13 Organic Acid Analyses

Before the analysis, the samples stored at −18°C were completely melted in a controlled temperature environment and then homogenized. The organic acid amounts of the samples were determined using an HPLC (Shimadzu Prominence). A 4 g sample was taken from the ice cream samples, and 20 mL of 0.01 N H_2_SO_4_ was added. Then, it was vortexed, passed through a 0.45‐μm filter, and injected into the system (Güzel Seydim et al. 2000). The system specifications used: CBM: 20ACBM, Detector: DAD (SPD‐M20A), Column Oven: CTO‐10ASVp, Pump: LC20 AT, Autosampler: SIL 20ACHT, Computer Program: LC Solution, Column: ODS 4 (250 mm × 4.6 mm, 5 μm) (GP Sciences, Inertsil ODS‐4, Japan), Mobile phase: ultrapure water adjusted to pH 3 with orthophosphoric acid (Aktaş et al. 2005). Before the analyzes, the HPLC was calibrated with 10 different organic acid standards and curves were plotted. The measurements were carried out in two parallel runs.

### 2.14 Determination of Fatty Acids

The fatty acid analyses of the samples were conducted utilizing a GC/MS (AGILENT 5975 C AGILENT 7890A GC). The device utilized the MSDCHEM software and a DB WAX column (50 × 0.20 mm, 0.20 μm). Prior to analysis, the samples preserved at −18°C were entirely melted in a regulated temperature setting and subsequently homogenized. The initial oven temperature was established at 80°C, and after a duration of 4 min, it was elevated to 175°C at a pace of 13°C per minute. It remained at this temperature for 27 min. Subsequently, it rose to 215°C at a pace of 4°C per minute. This temperature was maintained for 5 min. Subsequently, it escalated to 240°C at a pace of 4°C per minute. It remained at this temperature for 15 min. The temperature of the detector and injector is 240°C. The injection volume is established at 1 μL, with the detector and injector temperatures fixed at 240°C. In the studies, a 1.5 M concentration of HCl served as the derivatizing agent, with a derivatization temperature of 80°C and a duration of 2 h (Bardakçı and Secilmis 2006).

All physicochemical, textural, and microbiological studies (including first dripping time, meltdown time, overrun, texture parameters, pH, water activity, color, yeast counts, organic acids, and fatty acids) were conducted in duplicate for each sample group. Environmental conditions were meticulously checked and maintained consistently during the tests. Daily calibrations for instrumental analyses (pH meter, water activity device, colorimeter, HPLC, and GC/MS) were conducted using suitable standards in accordance with the makers' specifications prior to measurements.

### 2.15 Statistical Analysis

In this study, two‐way analysis of variance was used to determine the differences (*p* < 0.05) between the samples by double parallel and replicate. The impact of several yeast strains on the assessed parameters was established. All data underwent analysis of variance (ANOVA), succeeded by Duncan's multiple range tests to assess the differences between the means using SPSS software (version 28). The experimental design was entirely randomized with repetitions.

## 3 Result and Discussion

It has been determined that the interaction of sample type on the initial drop values of the physical properties of the ice cream samples is highly significant (*p* < 0.0001) (Table 1). The melting behavior of ice cream is defined as an empirical characteristic that reflects the melting resistance of ice cream when exposed to warm temperatures and is closely related to thermal conductivity, heat capacity, and the microstructure of the ice cream (Sun Waterhouse et al. 2013). The first dripping and meltdown times of the samples varied between 20.78–26.97 s and 86.78–97.30 s, respectively (Table 1). In samples produced by yeast inoculation, the first dripping and meltdown times were reduced compared to the control sample; on the upshot, different yeast inoculations in the ice cream mix have caused an extension in the initial dripping and complete melting times (*p* < 0.05). The highest increase was observed in samples with *S. cerevisiae* var. *boulardii* added, with values of 26.97 and 97.30 s. The addition of microorganisms to ice cream causes different effects on the melting properties of the ice cream mix, although the degree of this effect is not very significant (Alamprese et al. 2005).

**TABLE 1:** Physical analysis results of ice cream samples (per 2 kg ice cream sample).

Samples	Firt drip (s.)	Meltdown (s.)	Overrun
Control	26.97 ± 0.84^a^	97.30 ± 2.70^a^	50.76 ± 1.92^a^
*S. cerevisiae*	20.78 ± 1.88^b^	86.78 ± 2.31^b^	44.80 ± 0.88^b^
*S. cerevisiae* var. *boulardii*	22.49 ± 2.92^ab^	90.80 ± 2.34^b^	49.31 ± 1.68^a^
*K. marxianus* var. *lactis*	22.85 ± 2.11^ab^	89.35 ± 1.14^b^	41.21 ± 1.54^b^
*p* value	< 0.0001	0.145	0.11
*r*	0.382	0.380	−0.687

*Note:* ^a‐b^(↓): Values with the same capital letters in the same column for each analysis differ significantly (*p* < 0.05). *p* < 0.0001: Statistically too much significant.

The melting rate of ice cream can be determined by placing a sample of ice cream on a wire mesh sieve at high temperatures and measuring the rate of liquid accumulation underneath the container (Muse and Hartel 2004). As the ice cream melts, heat from the surrounding warm air is transferred to the ice cream, causing the ice crystals to melt. Initially, the ice melts on the outer part of the ice cream, creating a localized cooling effect (near the melting ice). The water from the melting ice needs to spread into the viscous, unfrozen serum phase, and this diluted solution then flows downward through the structural elements (stabilized fat globules, air cells, and remaining ice crystals) to drip from the screen (due to gravity). During melting, the flow of this diluted solution is initially over the outer part of the ice cream. When sufficient heat penetration causes the ice crystals inside the ice cream to melt, the diluted solution also begins to flow from the inside. The components within the mix (the amount of air, the structure and composition of the ice crystals, the structure of the fat, the composition of the curds, and other components of the mix, etc.) affect the time it takes for the heat generated outside to reach the inner parts (Zahorulko et al. 2021).

Ingredients that enhance the functional properties of ice cream (antioxidants, minerals, probiotics, prebiotics, plant proteins, and fibers) can indirectly affect its melting properties through changes in its microstructure. Specifically, shortening the initial dripping and complete melting times helps preserve these components (Akarca et al. 2024).

Ice cream with low overruns melt faster than ice cream with high overruns. This slower melting rate in ice creams with high overruns is due to the reduced heat transfer from the larger volume of air. Another factor that affects the distance the melted liquid needs to flow is the distance it takes to melt (Hwang et al. 2024). This statement supports our research findings (Table 1). Similar to our research findings, Kozłowicz et al. (2019) stated that the initial dripping and complete melting times of ice creams produced by fermenting the mix showed a decrease compared to the control samples.

Overrun can be defined as the measure of the amount of air added to the mix during the transformation stage of the mix into ice cream (Cruz et al. 2009). Overrun values of the samples varied between 41.21 and 50.76 (Table 1). Samples produced by inoculating the ice cream mix with yeast showed reduced the overrun values compared to the control sample (*p* < 0.05). Among the samples, the ones produced with the addition of *K. marxianus* var. *lactis* showed the most significant decrease in overrun value, followed by the samples produced with the addition of *S. cerevisae* and *S. cerevisiae* var. *boulardii*. In previous studies, Abghari et al. (2011), Sarwar et al. (2021), and Göktaş et al. (2022) have reported similar results. The yeasts added to the mixtures metabolized the sugars in the environment, leading to a decrease in mix viscosity and consequently a reduction in overrun amounts. Additionally, the decrease in pH also has negative effects on overrun (Allen et al. 2006).

In general, shortening the overrun period does not have a significant impact on functionality and nutritional value (Mayangsari et al. 2025). However, various studies have shown that probiotic viability is better preserved at lower overrun values compared to higher overrun values (Gao et al. 2021).

The firmness, consistency, cohesiveness, and index of viscosity values of the ice cream mix samples showed significant differences among all samples (*p* < 0.05; Table 2).

**TABLE 2:** Textural analysis results of mix of ice cream samples (per 2 kg ice cream sample).

Samples	Firmness (g)	Consitency (g s)	Cohesiveness (g)	Index of viscosity (g s)
Control	19.97 ± 1.27^a^	297.83 ± 17.09^a^	−11.96 ± 1.13^b^	−13.91 ± 2.67^b^
*S. cerevisiae*	13.71 ± 0.16^c^	251.21 ± 7.42^b^	−5.37 ± 1.24^a^	−2.38 ± 0.89^a^
*S. cerevisiae* var. *boulardii*	16.18 ± 0.81^b^	279.28 ± 0.39^ab^	−7.40 ± 0.77^a^	−9.88 ± 1.52^b^
*K. marxianus* var. *lactis*	13.97 ± 0.33^c^	270.65 ± 7.02^ab^	−7.19 ± 0.09^a^	−2.48 ± 0.48^a^
*p* value	0.04	0.40	0.008	0.05
*r*	0.132	−0.351	0.059	−0.082

*Note:* ^a‐b^(↓): Values with the same capital letters in the same column for each analysis differ significantly (*p* < 0.05). *p* < 0.01: Statistically too significant; *p* < 0.05: Statistically significant; *p* > 0.05: not statistically significant; ns, not statistically significant.

Firmness and consistency values are related to the structural properties of the ice cream mix (Freire et al. 2020). The firmness and consistency values of the mix samples decreased with the addition of different yeasts to the mix (*p* < 0.05). The greatest decrease, with values of 13.71 (g) and 251.21 (g s) respectively, occurred in the samples to which *S. cerevisiae* was added (Table 2).

Carbohydrates such as sucrose and lactose present in the mix have a positive effect on the water‐holding capacity of the mix. The fermentation process can lead to a reduction in available free water through various mechanisms. Yeast cells exhibit a high level of water retention due to their cellular structure, which can bind water and thus decrease aw (Öztürk‐Yalçın et al. 2024).

The cohesiveness and index of viscosity values of the ice cream mixes decreased with the addition of yeast (*p* < 0.05). The samples with the greatest decrease in cohesiveness and index of viscosity values were those with *S. cerevisiae* added, with values of −5.37 (g) and −2.38 (g s).

Rheology, the study of deformation and flow, is a fundamental physical property related to the processability of a material, primarily influenced by the molecular weight and size of its components (Li et al. 2020). The rheological behavior of the ice cream mix is much more complex than that of a simple liquid (Scholten 2013). Fermentation can produce organic acids and CO_2_, leading to a more fluid consistency of the mixture. Yeasts like *Kluyveromyces*, known for their lactose fermentation capacity, can also produce compounds that reduce viscosity while increasing emulsification capacity and the breakdown of larger fat globules (Boscaino et al. 2024).

Viscosity, which is the resistance of a liquid to flow, is defined as the internal friction that tends to resist the sliding of one liquid element over another. This frictional force has a significant impact on the emulsion formed within the mix. For liquids, viscosity is the most important element of rheology (Goff and Hartel 2013). Ice cream mix is an emulsion composed of four phases with a complex composition (cryoconcentrate, ice crystals, fat globules, and air) (Anjo et al. 2021). Especially, the hydrolysis of fat molecules due to the addition of yeast has reduced the emulsion stability and consequently the mix viscosity. Especially, the hydrolysis and fermentation of lactose and sucrose present in the mix have caused a decrease in these values of the samples.

It has been determined that the interaction of sample variety on the physical values of ice cream samples, specifically pH and aw, is highly significant (*p* < 0.0001) (Table 3). Different yeast inoculations in the ice cream mix have caused a decrease in pH and aw values (*p* < 0.05).

**TABLE 3:** Physicochemical, textural, and microbiological properties of ice cream samples (per 2 kg ice cream sample).

Samples	pH	a_w_	Firmness (g)	Yeast count (log cfu/g)
Control	6.52 ± 0.01^a^	0.730 ± 0.001^a^	32.40 ± 9.57^a^	2.41 ± 0.03^d^
*S. cerevisiae*	6.30 ± 0.01^d^	0.707 ± 0.001^ab^	34.79 ± 2.66^a^	7.09 ± 0.02^b^
*S. cerevisiae* var. *boulardii*	6.36 ± 0.02^c^	0.705 ± 0.001^b^	48.06 ± 6.41^a^	8.05 ± 0.09^a^
*K. marxianus* var. *lactis*	6.42 ± 0.02^b^	0.710 ± 0.001^b^	33.11 ± 2.15^a^	6.16 ± 0.06^c^
*p* value	< 0.0001	< 0.0001	0.157	< 0.0001
*r*	−0.303	−0.705	0.225	0.638

*Note:* ^a‐d^(↓): Values with the same capital letters in the same column for each analysis differ significantly (*p* < 0.05). *p* < 0.0001: Statistically too much significant, *p* < 0.01: Statistically too significant, *p* < 0.05: statistically significant, *p* > 0.05: not statistically significant, ns, not statistically significant.

It was determined that the pH values of the samples varied between 6.52 and 6.30, with the lowest pH value being in the sample with *S. cerevisiae* added and the highest pH value being in the control samples. The pH value of the ice cream mix is related to the composition of the mix. The lower pH values of the samples produced with the addition of three different yeasts, compared to the control sample. Yeast plays a crucial role in fermentation processes where it metabolizes sugars into ethanol and carbon dioxide, with subsequent oxidation forming organic acids, primarily acetic acid. In the context of ice cream, the fermentation initiated by yeast can produce lactic and acetic acids, which impact the flavor, acidity, and preservative qualities of the final product (Wyk et al. 2023). Similar to our research findings, Sarwar et al. (2021), Göktaş et al. (2022), Mahittikon et al. (2023) and Akarca et al. (2024) have stated that the addition of probiotic bacteria reduces the pH values of the samples.

Ice cream samples produced with yeast inoculation were found to have lower a_w_ values compared to the control sample (*p* < 0.05). In the ice cream mix samples, the water activity values ranged from 0.705 to 0.730 (Table 3), with the lowest a_w_ value found in the samples where *S. cerevisiae* was added to the mix. Akarca et al. (2024) found that the addition of lactic acid bacteria to the mix reduced the a_w_ value in a manner similar to our research results.

Yeasts contain cell walls rich in mannoproteins and polysaccharides that can interact with water and macromolecules in dairy systems, influencing water mobility and binding. The literature on yeast‐derived β‐glucans and parietal polysaccharides demonstrates that polysaccharide components can modify the rheology and moisture interactions in dairy and ice‐cream‐like matrices, affecting water distribution between bound and free water pools (Tomczyńska‐Mleko et al. 2024). These interactions can reduce the fraction of free, unbound water available in the system, contributing to a lower measured a_w_. In addition, yeast β‐glucans have been shown to form gel networks in stabilization systems, which can trap water and reduce its mobility, thereby lowering a_W_ relative to systems without such networks (Kawai et al. 2021).

The total yeast counts of the samples varied between 2.41 and 8.05 log cfu/g (Table 3). In the ice cream samples, the yeast counts varied according to the type of yeast added (*p* < 0.05). In ice cream production, yeast species were inoculated into the mixes at an average level of 10^7^–10^8^ log cfu/g. The mix was cooled to 37°C before inoculation, and the yeasts were inoculated. Then, the mixes were left to mature at 4°C for 24 h. The count of inoculated yeasts (7–8 Log cfu/mL) was determined using a densitometer (Biosan 1B, Türkiye) calibrated to the 0.5 McFarland standard (Akarca and Şevik 2021). As a result of the microbiological analyses conducted on the ice cream samples, the highest yeast count was detected in the samples supplemented with *S. boulardii* at 8.05 log cfu/g, followed by the samples supplemented with *S. cerevisiae* at 7.09 log cfu/g (Table 3).

*S. cerevisiae* shows optimal growth in the temperature range of 30°C–35°C (Tofighi et al. 2014). At this degree, *S. cerevisiae* var. *boulardii* and *K. marxianus* var. *lactis* grow well at 37°C (Nur Hossain et al. 2020; Petka Poniatowska et al. 2021). The minimum temperatures for yeast growth, despite showing very different variations in various studies, commonly indicate that generation times are prolonged at low temperatures. The low mixed temperature continued to increase the number of yeasts until it fell below the minimum threshold at which yeasts can develop. At lower temperatures, they can survive even if they cannot develop (Nonklang et al. 2008; López‐Malo et al. 2013).

In many studies conducted, although there are differences regarding the minimum number of probiotic microorganisms required for a food to be classified as a probiotic food, it has been reported that this number should be at least 7 log cfu/g‐mL (Butt et al. 2021; Abd‐Elmonem et al. 2022; Abdel‐Monem et al. 2022). In our study, among the ice cream samples produced with two different probiotic yeast additions, only *S. cerevisiae* was present. The presence of *S. cerevisae* var. boulardii at a level of 8 logs gives the ice cream a probiotic product characteristic.

One of the most important quality criteria of a food in the eyes of consumers is its color values (Chranioti et al. 2015). It has been determined that the interaction of sample type on the b* value of the ice cream samples is significant (*p* < 0.05) (Figure 1). It has been determined that the addition of yeast to the ice cream mix does not have any statistically significant effect on the L* and a* values of the products (*p* > 0.05), whereas it does have a statistically significant effect on the b* value (*p* < 0.05).

**FIGURE 2:**
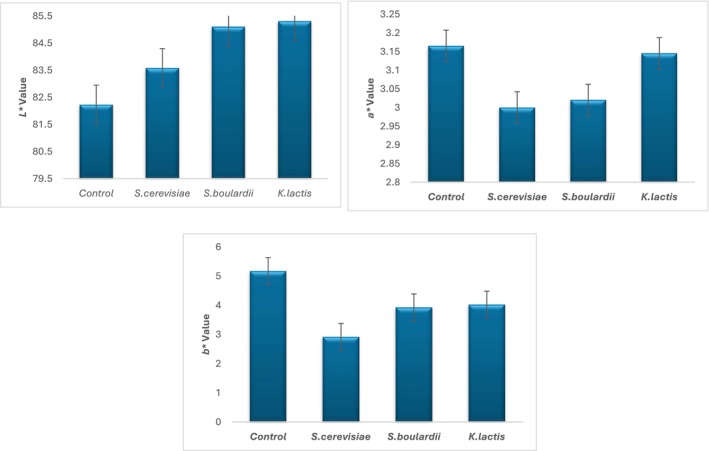
Color values of ice cream samples.

The L* values of the samples ranged from 85.31 to 82.23, the a* values from 3.17 to 3.00, and the b* values from 5.17 to 2.92 (Figure 2). Although the addition of yeast to the ice cream mix statistically showed that the changes in the a* values were insignificant, a decrease in red and yellow tones and an increase in green and blue tones were observed in the ice cream mixes. The changes in the color values of the samples during fermentation were caused by the conversion of nutrients (sugars) in the environment by yeasts into other related substances (organic acids, CO_2_, ethanol), which in turn led to changes in the color of the ice cream mix. Min Zhao et al. (2021) reported that the a* and b* values of their samples decreased because of fermentation.

Yeasts can alter color by absorbing pigments from the environment without completely removing the color from the system. More broadly, pigment absorption by yeast can shift color coordinates toward higher L values, making the product appear lighter, as the concentration of available colored pigments in the milk matrix decreases at the measurement site (McCullough et al. 2023). In ice creams produced by mixed yeast inoculation, metabolic byproducts and lactose/Maillard‐like reactions can affect color through lipid‐protein interactions affecting pigments and chromaticity. Reductions in a (redness) and b (yellowness) values may result from changes in pigment formation, pigment degradation, or light scattering properties of the ice cream emulsion influenced by microbial activity (Durmus 2020).

Among the organic acids detected in the ice cream samples, it was found that the interaction of sample variety had a very high effect on lactic, malic, oxalic, and formic acids (*p* < 0.0001), a significant effect on citric acid (*p* < 0.01), and a significant effect on malic acid (*p* < 0.05) (Table 5). The organic acid amounts (lactic, citric, oxalic, tartaric, and formic) in the samples increased with the addition of yeast, while the malic acid amounts decreased (*p* < 0.05). However, no changes were detected in the amounts of ascorbic acid. The organic acids with the highest increase were identified as formic, malic, and lactic acid in the samples produced with the addition of *S. cerevisiae* (Table 4).

**TABLE 4:** Organic acid content of ice cream samples (mg/kg) (per 2 kg ice cream sample).

Samples	Ascorbic acid	Lactic acid	Citric acid	Malic acid
Control	1.91 ± 0.008^a^	246.41 ± 13.41^c^	7.45 ± 0.08^c^	4.71 ± 1.18^d^
*S. cerevisiae*	1.91 ± 0.007^a^	569.67 ± 5.44^a^	7.77 ± 0.13^b^	49.00 ± 3.01^a^
*S. cerevisiae* var. *boulardii*	1.91 ± 0.001^a^	544.81 ± 8.23^a^	8.18 ± 0.04^a^	38.38 ± 2.18^b^
*K. marxianus* var. *lactis*	1.91 ± 0.004^a^	427.59 ± 29.37^b^	7.49 ± 0.07^c^	17.63 ± 0.60^c^
*p* value	0.814	< 0.0001	0.004	< 0.0001
*r*	−0.377	0.452	−0.440	0.181

Samples	Oxalic acid	Tartaric acid	Formic acid
Control	0.544 ± 0.01^c^	0.873 ± 0.03^b^	427,09 ± 13.09d
*S. cerevisiae*	8.960 ± 0.21^a^	1.066 ± 0.05^a^	1495,49 ± 16.87a
*S. cerevisiae* var. *boulardii*	2.925 ± 0.12^b^	1.024 ± 0.06^a^	1323.00 ± 43.32b
*K. marxianus* var. *lactis*	0.861 ± 0.03^c^	0.948 ± 0.05^ab^	981,15 ± 28.10c
*p* value	< 0.0001	0.05	< 0.0001
*r*	−0.168	0.251	0.408

*Note:* ^a‐d^(↓): Values with the same capital letters in the same column for each analysis differ significantly (*p* < 0.05). *p* < 0.0001: statistically too much significant, *p* < 0.05: statistically significant, *p* < 0.05: not statistically significant, ns, not statistically significant.

*S. cerevisiae* is noted for its robust fermentative capacity, which is crucial when incorporated into dairy products. It predominantly converts sugars into organic acids through fermentation processes, enhancing both flavor and preservative characteristics in the final product. The implication of *S. cerevisiae* as a superior fermentative agent is supported by various studies that emphasize its metabolic efficiency in producing higher concentrations of lactic and acetic acids, compared to other yeasts (Marcus et al. 2021; Galli et al. 2022; Ohstrom et al. 2023).

Fermentation is a process that helps large organic molecules break down into simpler molecules under the influence of microorganisms. Yeast enzymes convert sugars and starches into alcohol, while proteins are converted into peptides/amino acids (Melini et al. 2019; Sanlier et al. 2019). Yeasts have a wide variety of carbon sources they can metabolize (such as polyols, alcohols, organic acids, and amino acids), but they prefer sugars. Yeast has the capacity to metabolize hexoses (glucose, fructose, galactose, or mannose) and disaccharides (maltose or sucrose) as well as two‐carbon compounds (ethanol or acetate). The metabolic pathways used by yeast are Embden‐Meyerhof glycolysis, the tricarboxylic acid cycle (TCA), the pentose phosphate pathway, and oxidative phosphorylation. Using these metabolic pathways, they produce ethanol, CO_2_, and various organic acids. Organic acids are the primary products of yeast carbohydrate metabolism (Zailani and Adnan 2022).

The notable disparities in organic and fatty acid profiles across the yeast strains highlight their unique metabolic processes. The elevated production of organic acids and accumulation of saturated fatty acids in samples inoculated with *S. cerevisiae* , as opposed to *S. boulardii* and *K. lactis*, arise from intrinsic differences in carbon source utilization, regulatory networks controlling glucose repression, and lipid biosynthesis pathways in dairy environments (Koivistoinen et al. 2013; Nussio 2024). *S. cerevisiae* exhibited the most potent fermentative activity, resulting in significant chemical modifications, but *S. boulardii* notably preserved the maximum probiotic viability without substantially affecting the physical characteristics of the ice cream matrix relative to the control group.

These strain‐specific biochemical and physical interactions determine the final texture and shelf stability while directly augmenting the functional and nutritional content of the ice cream. Thus, using specialized yeast strains in ice cream formulation enables the precise enhancement of its health‐promoting properties, converting a conventional dessert into a functional meal with unique biological advantages.

It has been determined that the interaction of sample variety has a highly significant effect (*p* < 0.0001) on the amounts of all fatty acids in the ice cream samples except for pentadecanoic, palmitic, palmitoleic, stearic, linolenic, and eicosanoic acids, a significant effect (*p* < 0.01) on the amounts of pentadecanoic, stearic, and eicosanoic acids, and a significant effect (*p* < 0.05) on the amount of palmitic acid. Additionally, the sample variety showed a positive correlation at the *p* < 0.01 level with butanoic, lauric, and myristic acids, and a positive correlation at the *p* < 0.05 level with caproic, caprylic, capric, undecanoic, pentadecanoic, palmitic, and palmitoleic acids. Additionally, the interaction of sample variety showed a highly correlative effect in a negative direction at the *p* < 0.05 level on stearic and oleic acids (Table 5).

**TABLE 5:** Distribution of % fatty acids of ice cream samples (per 2 kg ice cream sample).

	Fatty acids				
	Butanoic acid (C4.0)	Caproic acid (C6.0)	Caprylic acid (C8.0)	Capric acid (C10.0)	Undecanoic acid (C11.0)
RT (s.)	7.476	12.439	20.797	31.494	42.588
Control	0.129 ± 0.01^d^	0.180 ± 0.01^c^	0.215 ± 0.01^b^	0.624 ± 0.03^b^	0.816 ± 0.01^c^
*S. cerevisiae*	0.443 ± 0.01^c^	0.536 ± 0.01^b^	0.469 ± 0.03^a^	1.182 ± 0.02^a^	1.595 ± 0.01^b^
*S. cerevisiae* var. *boulardii*	0.504 ± 0.01^b^	0.583 ± 0.01^a^	0.466 ± 0.03^a^	1.164 ± 0.01^a^	1.667 ± 0.03^a^
*K. marxianus* var. *lactis*	0.576 ± 0.02^a^	0.577 ± 0.01^a^	0.459 ± 0.02^a^	1.177 ± 0.01^a^	1.646 ± 0.02^ab^
*p* value	< 0.0001	< 0.0001	< 0.0001	< 0.0001	< 0.0001
*r*	0.918**	0.824*	0.744*	0.768*	0.804*

	Fatty acids				
	Lauric acid (C12.0)	Myristic acid (C14.0)	Pentadecanoic acid (C15.0)	Palmitic acid (C16.0)	Palmitoleic acid (C16.1)
RT (s.)	58.053	53.240	54.701	63.185	63.884
Control	0.404 ± 0.02^c^	5.013 ± 0.06^d^	0.342 ± 0.01^b^	52,949 ± 0.25^b^	0.845 ± 0.06^a^
*S. cerevisiae*	0.643 ± 0.01^b^	7.875 ± 0.09^c^	0.579 ± 0.01^a^	59,384 ± 1.04^a^	1.072 ± 0.01^a^
*S. cerevisiae* var. *boulardii*	0.729 ± 0.03^a^	6.066 ± 0.05^b^	0.655 ± 0.07^a^	57,813 ± 3.29^a^	1.139 ± 0.02^a^
*K. marxianus* var. *lactis*	0.746 ± 0.05^a^	6.321 ± 0.03^a^	0.572 ± 0.01^a^	55,861 ± 2.95^a^	1.605 ± 0.58^a^
*p* value	< 0.0001	< 0.0001	0.003	0.026	0.213
*r*	0.900**	0.843**	0.717*	0.772*	0.759*

	Fatty acids		
	Stearic acid (C18.0)	Oleic acid (C18.1)	Linoleic acid (C18.2)
RT (s.)	72.022	72.677	72.867
Control	6300 ± 0.22^a^	30,037 ± 0.21^a^	0.449 ± 0.02^c^
*S. cerevisiae*	5213 ± 0.13^bc^	16,964 ± 0.08^b^	0.607 ± 0.02^b^
*S. cerevisiae* var. *boulardii*	5480 ± 0.18^b^	15,546 ± 0.30^c^	0.495 ± 0.03^a^
*K. marxianus* var. *lactis*	4982 ± 0.05^c^	15,152 ± 0.20^d^	0.572 ± 0.03^b^
*p* value	0.005	< 0.0001	< 0.0001
*r*	−0.807*	−0.808*	0.412

	Fatty acids		
	γ‐Linolenic acid (C18.2)	Linolenic acid (C18.3)	Eicosanoic acid (C20.0)
RT (s.)	73.063	74.270	80.121
Control	0.112 ± 0.01^b^	0.425 ± 0.06^a^	0.485 ± 0.02^c^
*S. cerevisiae*	0.925 ± 0.06^a^	0.550 ± 0.06^a^	0.560 ± 0.03^b^
*S. cerevisiae* var. *boulardii*	0.105 ± 0.01^b^	0.570 ± 0.04^a^	0.640 ± 0.03^a^
*K. marxianus* var. *lactis*	0.885 ± 0.05^a^	0.455 ± 0.05^a^	0.460 ± 0.01^c^
*p* value	< 0.0001	0.129	0.005
*r*	0.419	0.171	0.008

Samples	SFA	USFA	SCFA	MCFA	LCFA
Control	67,457	31,868	2368	59,149	37,808
*S. cerevisiae*	78,479	20,118	12,743	61,035	24,819
*S. cerevisiae* var. *boulardii*	75,767	17,855	11,179	59,607	22,836
*K. marxianus* var. *lactis*	73,380	18,669	11,502	58,038	22,506

*Note:* ^a‐f^(↓): Values with the same capital letters in the same column for each analysis differ significantly (*p* < 0.05). ** is significant at the 0.01 level (two‐tailed), * Correlation is significant at the 0.05 level (two‐tailed), *p* < 0.0001: statistically too much significant, *p* < 0.01: statistically too significant, *p* < 0.05: Statistically significant, *p* > 0.05: not statistically significant, ns, not statistically. * *p* < 0.05. ** *p* < 0.01. significant.

Abbreviations: B.Ag, before aging; RT, retention time; CLA, conjugated linolenic acid; LCFA, long chain fatty acids (≥ C:18. A.Ag, after aging); MCFA, medium chain fatty acids (C:14‐C:16); SCFA, short chain fatty acids (C:4‐C:12); SFA, satured fatty acids; USFA, unsatured fatty acids.

The amounts of fatty acids varied depending on the addition of yeast (*p* < 0.05). When examining the distribution of the identified fatty acids, it was found that in all samples, the amount of saturated fatty acids was higher than that of unsaturated fatty acids, and the amount of medium‐chain fatty acids was higher than that of short‐ and long‐chain fatty acids. The highest saturated fatty acid content, 78.479%, was found in samples produced with the addition of *S. cerevisiae*, while the lowest saturated fatty acid content, 67.457%, was found in the control samples. The highest medium‐chain fatty acid was determined to be 61.035% in the product produced with the addition of *S. cerevisiae*.

Ice cream produced with *S. cerevisiae* inoculation exhibits a higher saturated fat content compared to *S. boulardii* and *K. lactis*, which can be attributed to strain‐specific milk matrix interactions affecting fat emulsification, droplet stability, and crystallization during processing and storage. Although *S. boulardii* and *S. cerevisiae* are genetically very close, they exhibit different physiological and stress response behaviors, which can lead to different interactions with food matrices (Degirmencioğlu et al. 2016; Pais et al. 2020).

In this study, the probiotic classification was based on yeast viability determined immediately after production. Although the initial counts met the probiotic threshold (≥ 7 log cfu/g), the lack of viability monitoring throughout the storage period is a limitation of the present work. Future studies should investigate the survival and stability of these yeast strains during the extended shelf life of the ice cream.

However, the substantial magnitude of these variations—particularly the significant reduction in oleic acid and the increase in saturated fatty acids within a 24‐h low‐temperature fermentation—suggests that yeast metabolic activity alone may not fully account for these differences. Despite efforts to standardize raw materials, inherent batch‐to‐batch natural variability in the fat source (e.g., seasonal variations in milk fat composition or kaymak properties) likely contributed to the observed fatty acid profile fluctuations.

## 4 Conclusion

In this study, the changes in the physicochemical and nutritional properties of ice cream caused by three different yeasts inoculated into the ice cream mix, as well as whether the product gains probiotic properties depending on the number of added probiotic yeasts, were determined.

The addition of yeast to the ice cream mix has caused the first drip, complete melting, overrun, firmness, consistency, pH, and a_w_ values to decrease. Additionally, while no change was detected in the ascorbic acid values of the organic acid amounts in the samples produced with yeast addition, an increase in other acids was observed. The organic acids with the highest increase were determined to be formic (1495.49 mg/kg), malic (49.00 mg/kg), and lactic acid (569.67 mg/kg) in the samples produced with the addition of *S. cerevisiae* . Additionally, the amounts of fatty acids in the ice cream samples varied depending on the addition of yeast. In the samples produced with yeast addition, the amounts of unsaturated fatty acids decreased (lowest 17.855%), while the amounts of saturated fatty acids increased (highest 78.479%). Additionally, the amounts of short and medium‐chain fatty acids increased compared to the control sample, while the amounts of long‐chain fatty acids decreased (Table 4).

Ice cream is a dairy product that is loved by everyone around the world, especially children, and is consumed in large quantities. Thanks to its high nutritional value, it is also considered an important food item. In addition to all these effects of ice cream, the production of the mix after fermentation with yeast inoculation has also led to an increase in the functional and nutritional properties of the obtained products. Additionally, the presence of *S. cerevisiae* var. *boulardii* at a level of over 8 log cfu/g has also given the ice cream a probiotic product characteristic.

Today's consumers increasingly demand foods with improved functional properties. As a result of fermenting the ice cream mixture with different yeasts, it has become richer in terms of nutritional values as well as physicochemical and functional properties. Especially in ice creams produced with yeast inoculation, the higher amounts of short‐chain fatty acids and γ‐linoleic acid reveal the importance of the product in terms of nutrition and health.

## Author Contributions

**Ayşe Janseli Denizkara:** conceptualization, methodology, resources, writing – review and editing. **Gökhan Akarca:** conceptualization, data curation, methodology, resources, writing – original draft, validation. **Mehmet Kilinç:** conceptualization, funding acquisition, project administration.

## Ethics Statement

The authors have nothing to report.

## Consent

The authors have nothing to report.

## Conflicts of Interest

The authors declare no conflicts of interest.

## Data Availability

The original data with the respective analysis corresponding to the results shown in this work are available up to reasonable requirements.
